# Candidate Genes for Expansion and Transformation of Hematopoietic Stem Cells by NUP98-HOX Fusion Genes

**DOI:** 10.1371/journal.pone.0000768

**Published:** 2007-08-22

**Authors:** Lars Palmqvist, Nicolas Pineault, Carina Wasslavik, R. Keith Humphries

**Affiliations:** 1 Terry Fox Laboratory, British Columbia Cancer Agency, Vancouver, British Columbia, Canada; 2 Institute of Biomedicine, Sahlgrenska University Hospital, Göteborg, Sweden; 3 Departments of Medicine, University of British Columbia, Vancouver, British Columbia, Canada; Massachusetts General Hospital, United States of America

## Abstract

**Background:**

Hox genes are implicated in hematopoietic stem cell (HSC) regulation as well as in leukemia development through translocation with the nucleoporin gene *NUP98*. Interestingly, an engineered NUP98-HOXA10 (NA10) fusion can induce a several hundred-fold expansion of HSCs *in vitro* and NA10 and the AML-associated fusion gene NUP98-HOXD13 (ND13) have a virtually indistinguishable ability to transform myeloid progenitor cells *in vitro* and to induce leukemia in collaboration with MEIS1 *in vivo*.

**Methodology/Principal Findings:**

These findings provided a potentially powerful approach to identify key pathways mediating Hox-induced expansion and transformation of HSCs by identifying gene expression changes commonly induced by ND13 and NA10 but not by a NUP98-Hox fusion with a non-DNA binding homedomain mutation (N51S). The gene expression repertoire of purified murine bone marrow Sca-1+Lin- cells transduced with retroviral vectors encoding for these genes was established using the Affymetrix GeneChip MOE430A. Approximately seventy genes were differentially expressed in ND13 and NA10 cells that were significantly changed by both compared to the ND13(N51S) mutant. Intriguingly, several of these potential Hox target genes have been implicated in HSC expansion and self-renewal, including the tyrosine kinase receptor *Flt3*, the prion protein, *Prnp*, hepatic leukemia factor, *Hlf* and Jagged-2, *Jag2*. Consistent with these results, *FLT3*, *HLF* and *JAG2* expression correlated with HOX A cluster gene expression in human leukemia samples.

**Conclusions:**

In conclusion this study has identified several novel Hox downstream target genes and provides important new leads to key regulators of the expansion and transformation of hematopoietic stem cells by Hox.

## Introduction

The 39 clustered Hox proteins are an evolutionary preserved family characterized by a 60 amino acid DNA-binding motif called the homeodomain. *Hox* genes of the A, B and C but not D clusters are transcribed during normal hematopoiesis with their expression being confined to the immature subpopulations. Elimination or enforced expression of certain clustered *Hox* genes in mouse models has demonstrated profound effects on hematopoietic differentiation and persistent expression of normal or mutated Hox proteins in hematopoietic progenitors can result in leukemia in both mice and humans [1], [2]. *Hox* genes have also been linked to leukemia by virtue of their involvement in leukemia-specific translocations. Clustered *Hox* genes have repeatedly been identified in acute myeloid leukemia (AML) harboring translocations that generate novel fusion proteins containing the N terminal region of the nucleoporin gene, *NUP98*, and the C terminal Hox region including the homeodomain (HD). The involvement of homeobox genes as partners of NUP98 is of particular interest given the growing evidence linking *Hox* genes, particularly members of the 5′-located members of the HOXA cluster, such as *HOXA9*, to leukemia. To date at least seven clustered Hox genes have been found fused to *NUP98* in human leukemia, interestingly only from the Abd-B clustered *Hox* [3]–[11]. Furthermore, two non-clustered homeodomain-containing genes *PMX1* and *PMX2* have also been identified as *NUP98* fusion partners in de novo AML and t-AML respectively [12], [13].

The mechanisms by which Hox proteins mediate their effects and how they perturb cellular functions are not well understood. They seem to be highly context dependent in their actions [14] and in normal hematopoiesis their expression is tightly regulated [15]. Expression analysis and gain- or loss- of function studies have shown that Hox proteins play an important role in the regulation of early stages of hematopoiesis, including the self-renewal of hematopoietic stem cells and early progenitors [16]. DNA site-selection studies indicate that the homeodomain by itself has limited target sequence recognition. Additional binding specificity and stability is in part achieved through interaction with other homeodomain-containing proteins from the multimember PBX or MEIS1 families [17] and co-transduction of *MEIS1* with *Hox* and *NUP98-Hox* genes also strongly accelerate the onset of leukemia in mice [18]–[20]. There is also a considerable redundancy between different NUP98-Hox fusions in effects when expressed in bone marrow cells *in vitro* and their ability to collaborate with MEIS1 *in vivo*. An engineered NUP98-HOXA10 (NA10) fusion and the AML-associated fusion gene NUP98-HOXD13 (ND13) have a virtually indistinguishable ability to transform myeloid progenitor cells in vitro and to induce leukemia in collaboration with MEIS1 in vivo [21]. Furthermore, it has been shown that a N51S mutation in the DNA binding homeodomain abolishes the DNA binding ability of several *Hox* genes [22] and, indeed, the AML-associated fusion gene *NUP98-HOXD13 (ND13)* fusion also loses its leukemic effect from this mutation [19].

The relatively long latency of Hox-induced AML in mouse models strongly indicates that additional genetic events are required for full leukemic progression [19], [23], [24]. Indeed, data supports that Hox-containing fusions, as for most transcription factor-containing fusion oncogenes, alter the growth and differentiation of early hematopoietic precursors leading to the establishment of a preleukemic population of cells that are then susceptible to the acquisition of cooperating mutations [25]. In concordance with such a model, we recently demonstrated that constitutive expression of ND13 or NA10 was sufficient to induce a pre-leukemic state in primary bone marrow (BM) cells after extended *in vitro* culture. Though these cells had short-term repopulating potential, they were for the most part incapable of inducing AML on their own but could readily be converted into AML-inducing cells when complemented with MEIS1 or other genes [26]. Intriguingly, in short term culture, NA10 can promote high level expansion of long term repopulating cells; moreover a NUP98-fusion restricted to the homeodomain of HOXA10 induces similar levels of expansion of HSC [27]. Together, these findings suggest that NUP98-Hox fusions genes impact on crucial genetic programs involved in stem cell self-renewal and proliferation that may also contribute to leukemic transformation.

In an effort to gain insight into the nature of the possible genetic programs impacted by Hox relevant to stem cell self-renewal and leukemogenesis we have used microarray technology to assess gene expression perturbations induced by NUP98-HOX fusions 24 hours post transduction into murine primary bone marrow Sca-1+Lin- cells enriched in HSC and progenitors cells. We compared two NUP98-HOX fusions (NA10 and ND13) with a non DNA-binding and non-transforming ND13(N51S) homeodomain mutant. Surprisingly, a relatively small number of genes were significantly differentially expressed in cells harboring the ND13 or NA10 fusions compared to control cells and several potential target genes implicated in HSC expansion and self-renewal were identified with this approach.

## Materials and Methods

### cDNA constructs and retroviral vectors

The NUP98-HOXA10 and NUP98-HOXD13 fusion gene as well as the N51S-ND13 dead homeodomain mutant gene constructs have been described elsewhere [19]. In short, the cDNAs were subcloned into the murine stem-cell virus (MSCV) 2.1 vector upstream of the internal ribosomal entry site (IRES) sequence linked to the gene encoding the enhanced green fluorescence protein (EGFP; Clontech). As a control, the MSCV vector carrying only the IRES-GFP cassette (GFP virus) was used. Production of high-titer, helper-free retrovirus was carried out by standard procedures [28]. Constructs have been validated by sequencing and correct expression and transmission were confirmed by western blot and Southern blot analysis.

### Retroviral infection of primary bone marrow cells

Mice were bred and maintained at the British Columbia Cancer Research Centre animal facility. Donors of primary BM cells were older than 12-weeks (C57Bl/6Ly-Pep3b×C3H/HeJ) F1 (PepC3) mice. Primary mouse BM cells were transduced as previously described [29]. Briefly, BM cells were harvested from mice treated 4 days previously with 150 mg/kg 5-fluorouracil (Faulding, Underdaler, Australia) and prestimulated for 48 hours in Dulbecco modified Eagle medium (DMEM) supplemented with 15% fetal bovine serum (FBS), 10 ng/mL human interleukin-6 (hIL-6), 6 ng/mL murine interleukin-3 (IL-3), and 100 ng/mL murine stem cell factor (mSF) (StemCell Technologies, Vancouver, BC, Canada). Cells were infected by co-cultivation with irradiated (4000 cGy x-ray) GP+E86 viral producer cells with the addition of 5 µg/mL protamine sulfate (Sigma, Oakville, ON, Canada). Loosely adherent and nonadherent cells were harvested from the co-cultures after 2 days and were cultured for 24 hours in the same medium without protamine sulfate.

### Cell sorting and harvest

The single cell suspensions collected were blocked for 10 min on ice with 5 µg/ml anti mouse CD16/CD32 (Fc Block, BD Pharmingen) in Phosphate Buffered Saline (STI)+2% Fetal Bovine Serum (PF). Cells were washed once with PF and then incubated on ice for 20 min with the primary mAb. Cells were then washed once, incubated with the secondary antibody if needed, washed again, and then analysed by flow cytometry using a FACSCalibur™ flow cytometer and CELLQuest™ software (BD Pharmingen). GFP+ Sca-1+Lin- cells were sorted using a FACSVantage™ (BD Pharmingen). Purity>90% were confirmed by re-analysis of sorted cells. The forward versus side scatter profile was used to gate on viable cells and an unstained sample was used to determine appropriate gating for expression. Monoclonal antibodies (mAbs) were all purchased from PharMingen (San Diego, CA) (phycoerythrin [PE]–labeled Gr-1, B220, Ter-119, CD4, CD5 and CD8).

### RNA extraction and array hybridization

Sorted cells were lyzed in Trizol™ (Invitrogen) and total RNA was extracted according to the manufacturer instructions. One hundred ng of total RNA from each sample were then double linear amplified with the ENZO BioArray High Yield RNA Transcript Labeling kit and the GeneChip Eukaryotic Small Sample Target Labeling Assay, Version II protocol (Affymetrix, Santa Clara, CA) to produce target for hybridization to Affymetrix MOE430 according to the manufacturer's instructions and performed at the Genome Science Centre, BC Cancer Agency, Vancouver, Canada. All experiments were performed in biological triplicate.

### Gene array analysis

Gene array data (CEL-files) were imported into GeneSpring® software version 7.3 (Silicon Genetics, Redwood City, CA). The GC-RMA method [30] was used for normalization and data was processed as follows: all values below 0.01 were set to 0.01, all of the genes in each sample were divided by the median of the specified list of 100 positive control genes present on the MOE430 chip and all samples were then normalized against the median of the MIG control samples. Each measurement for each gene in those specific samples was divided by the median of that gene's measurements in the corresponding control samples. We judged genes to be differentially expressed when the difference in expression in the ND13 and NA10 condition vs. the GFP control or the non-leukemic ND13(N51S) condition was at least 50%; and the extent of difference in expression was significantly different in the Student's t-test (p<0.05). Classification of genes into functional categories and to analyze signaling pathways was done by collecting annotations and keywords with the Onto-Express software [31] (http://vortex.cs.wayne.edu/ontoexpress), Affymtrix NetAffx (http://www.affymetrix.com/analysis/index.affx) and the Gene Ontology Tool and KEGG maps included in the GeneSpring 7.3 Software. Unigene and RefSeq IDs were used in the analysis to exclude redundant genes included in the array probe sets. The array data is deposited at Gene Expression Omnibus (GEO), (http://www.ncbi.nlm.nih.gov/geo) and the MIAME (minimal information about a microarray experiment) guidelines was followed for data presentation.

### Quantitative RT-PCR validation of murine bone marrow cells

Non-amplified RNA from the transduced murine primary bone marrow samples was used for validation with quantitative RT-PCR (qRT-PCR). RNA was isolated using Trizol™ and the samples were then treated with DNase I (amplification grade, Invitrogen). Complementary DNA (cDNA) was generated by reverse transcription (RT) with the iScript cDNA Synthesis Kit (BioRad Inc., Hercules, CA). Gene transcripts were quantified by real-time PCR using the iCycler apparatus (Bio-Rad Inc., Hercules, CA) and were detected with SYBR Green as flurochrome (IQ™ SYBR® Green Supermix, BioRad Inc.). Gene sequences for primer design were obtained from the NCBI Reference Sequences database (http://www.ncbi.nlm.nih.gov/RefSeq/). Primers were chosen using the Primer3 software (http://www.broad.mit.edu/cgi-bin/primer/primer3_www.cgi) and the specificity of all primer pairs was tested with electronic PCR using the mouse genome and the mouse transcript database (http://www.ncbi.nlm.nih.gov/sutils/e-pcr/reverse.cgi). The relative expression changes were determined with the 2^−ΔΔCT^ method [32] and the housekeeping glyceraldehyde-3-phosphate dehydrogenase (GAPDH) gene transcript was used to normalize the results. Primer sequences (5′ to 3′); *Pbx1*(NM_008783) forward primer ATCGGGGACATTTTACAGCA and reverse primer AGGCTTCATTCTGTGGCAGT; *Pbx2* (NM_017463) TGAAGCAAAGCACCTGTGAG and AGTGGCCTGTTTGCTGAAGT; *Pbx3* (NM_016768) AGAGCCAAATTGACCCAGAT and ATGGGACGCGTTCTACTCTG; *HoxA5* (NM_010453) CGCAAGCTGCACATTAGTCA and AGGTAGCGGTTGAAGTGGAA; *HoxA7* (NM_010455) GAAGCCAGTTTCCGCATCTA and CGTCAGGTAGCGGTTGAAAT; *HoxA9* (NM_010456) ACAATGCCGAGAATGAGAGC and GTTCCAGCGTCTGGTGTTTT; *HoxB4* (NM_010459) CTGGATGCGCAAAGTTCAC and TCCTTCTCCAACTCCAGGAC; *Meis1* (NM_010789) GCACAGGTGACGATGATGAC and AGGGTGTGTTAGATGCTGGAA, *Meis2* (NM_010825) AACGACGCCTTGAAAAGAGA and GCTCGCACTTCTCAAAAACC; *Meis3* (NM_008627) CAGCGACAGCTTGAAGAGAG and GCCAGCTCACACTTCTCAAA; *Flt3* (NM_010229) ATCCTTCCCCAACCTGACTT and TTGCCACCCATGTTCTGATA; *Evi1* (NM_007963) GGAGGAGGACTTGCAACAAA and GACAGCATGTGCTTCTCCAA; *Anxa1* (NM_010730) CACAGAGCCACCAGGATTTT and CGTTCGGAAATTGACATGAA; *Tgfbi* (NM_009369) GGAAGCTTCACCATCTTTGC and ATGTTGACGTTGCTCACCAG; *c-Jun* (NM_010591) TCCCCTATCGACATGGAGTC and TTTTGCGCTTTCAAGGTTTT; *Csf2rb* (NM_007781) CCTGGAACAAGGGAAGTTCA and CAATGCAGGCTGTAGCTGTC; *Ptprf* (NM_011213) TGGCCATCTCTTCATTAGGC and ACAGGCTCGGTATTTCCAGA; *Gapdh* (NM_008084) AACTTTGGCATTGTGGAAGG and ATGCAGGGATGATGTTCTGG.

### Patient samples

The part of the study involving patient samples and healthy volunteers was performed in accordance to the Declaration of Helsinki and with approval by the local ethics committee at Göteborg University and informed written consent was obtained from all participants. All samples were collected at diagnosis between year 2000 and 2006 and stored at the department of Clinical Chemistry and Transfusion Medicine at Sahlgrenska University Hospital. The analysis included 34 adult patients, 20 females and 14 males, with de novo AML, representing FAB subclasses M0-M5. Mean age at diagnosis was 56 year (range 26 to 83). Four healthy volunteers were donors of normal bone marrow that was pooled for assay normalization.

### TaqMan® Low density array (TLDA)

RNA from patient and healthy volunteer samples was isolated using Trizol™ (Invitrogen, Cat.No. 15596-026). Complementary DNA (cDNA) was generated from 500 ng RNA by reverse transcription (RT) with random primers and the Superscript II enzyme and RNase inhibitor (Invitrogen) in a reaction volume of 20 µL. The RT reaction was incubated at 42°C for 50 minutes followed by 15 minutes at 70°C. Before the enzymes were added the mix was preheated at 65°C for 10 minutes. All assays were performed on an ABI Prism 7900 HT real-time PCR-system with ABI SDS Software 2.2.3 (Applied Biosystems, Foster City, CA, USA). For the TLDA 20 µL of cDNA, corresponding to 25 ng starting RNA, was mixed with 30 µL water and 50 µL TaqMan Universal PCR Master Mix (Applied Biosystems, Stockholm, Sweden); 100 µL was loaded per port. Thermal cycling conditions were 50°C for 2 minutes, 94.5°C for 10 minutes, 97°C for 30 seconds and 59.7°C for 1 minute. The relative expression changes were determined with the 2^−ΔΔCT^ method [32] and the housekeeping beta-glucuronidase (GUSB, Hs99999908_m1) gene transcript was used to normalize the results. The following genes were analyzed (TaqMan assay ID number is indicated, Applied Biosystems); *HOXA5* (Hs00430330_m1), *HOXA7* (Hs00600844_m1), *HOXA9* (Hs00365956_m1), *HOXA10* (Hs00538183_m1), *PBX1* (Hs00231228_m1), *PBX2* (Hs00855025_s1), *PBX3* (Hs00608415_m1), *MEIS1* (Hs00180020_m1), *MEF2C* (Hs00231149_m1), *JAG2* (Hs00171432_m1), *PRNP* (Hs00175591_m1), *DDX4* (Hs00251859_m1) and *HLF* (Hs00171406_m1).

### Statistical analysis

Microsoft^®^ Excel in combination with the Excel plug-in software Analyse-It^®^ v1.73 was used for the statistical calculations. Pearson regression was used for comparison between gene array and qRT-PCR results. Spearman rank correlation was used to test possible associations. Non-parametric Kruskal-Wallis 1-way ANOVA were used to evaluate differences between groups.

## Results

### Transduction of murine primary bone marrow

Adult murine bone marrow cells transduced with vectors carrying ND13, NA10, ND13(N51S) or an empty GFP control vector were isolated on the basis of GFP expression by FACS 24 hours post-transduction. Viable transduced cells were further enriched for primitive hematopoietic cells by exclusion of cells expressing linage markers (Gr-1, B220, Ter-119, CD4, CD5 and CD8) and selection for cells expressing the stem cell antigen-1 (Sca-1). This resulted in an overall recovery of 0.25–6% of all cells with a purity of 90–95% for GFP expression. The cells were kept on ice during sorting and immediately lyzed in Trizol for RNA extraction. Three independent experiments were performed for each of the four different conditions included in the study.

### Gene array analysis and validation

**Figure 1:**
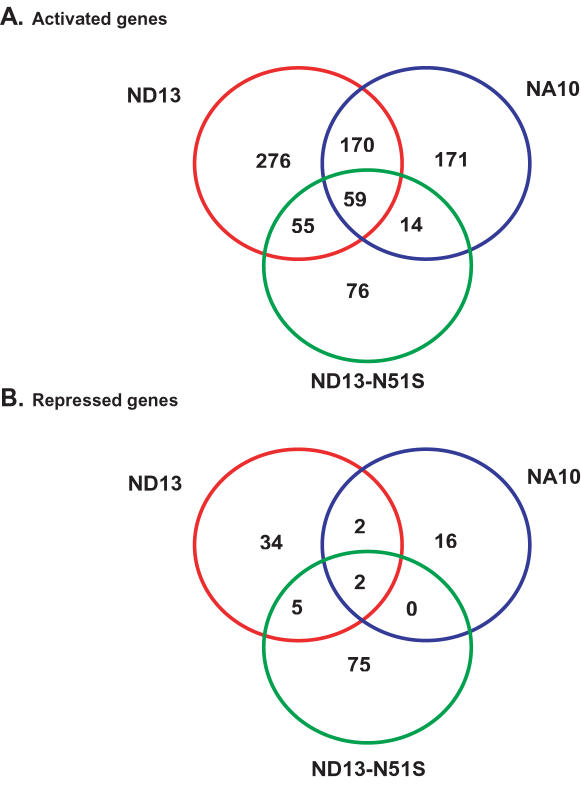
Venn diagram of genes significantly activated (A) or repressed (B) in Sca1+, Lin- BM cells expressing the ND13, NA10 or ND13(N51S) mutant fusion genes compared to GFP control.

After extraction, RNA was amplified and analyzed using the Affymetrix GeneChip MOE430A array containing 23,000 probe sets. The Gene Chip robust multi-array analysis (GC-RMA) was used for initial normalization and the GFP control was used for per gene normalization. Pearson correlation coefficient between the experimental replicates ranged between r = 0.92–0.99 suggesting low inter-experimental variation and the 3′-to-5′ ratios for Gapdh and Actin in all samples were less than 3.0 (ranging from 1.7–2.7), indicating that no serious bias was introduced by the RNA amplification procedure. Our main interest was to define the subset of genes that could explain the transforming and cell expansion potential of NUP98-HOX fusions in the Sca-1+, GFP+, Lin- cell population and that also could be direct NUP98-Hox binding target genes. First, all genes that were significantly differentially expressed between ND13, NA10 or the presumably non DNA-binding ND13(N51S) and the GFP control were identified. Genes were considered differentially expressed if they had a change in expression level compared to control of at least 50% and the extent of difference in expression was statistically significant (p<0.05) in a parametric Welsh-ANOVA t-test. More genes were activated than repressed by all NUP98-HOX fusions, 560 activated and 43 repressed genes with ND13, 414 activated and 20 repressed genes with NA10 and 204 activated and 82 repressed genes with ND13(N51S), (Figure 1). ND13 and NA10 had relatively more activation compared to the ND13(N51S) mutant and there was only a significant overlap on activated genes between ND13 and NA10 (Figure 1). Furthermore, of the 170 differentially expressed genes that were induced by both ND13 and NA10, 74 of these genes also differed significantly between ND13 and NA10 and the functionally inert ND13(N51S) mutant genes with at least a 50% difference in expression level (Table 1). These results suggest that ND13 and NA10 mainly act as transcriptional activators in undifferentiated BM cells and that this effect is dependent on an intact homeodomain. Moreover, the gene array results also indicate that a relatively small number of activated genes can be linked to NUP98-Hox induced effects on primitive hematopoietic cell function.

**Table 1:** Genes changed by NA10 and ND13 but not by the ND13(N51S) mutant compared to the GFP control.

Gene name and description	Accession #	ND13 fold change	ANOVA p-value	NA10 fold change	ANOVA p-value	Biological process
Crisp1, cysteine-rich secretory protein 1	NM_009638	18.3	6.04E-06	30.8	7.92E-06	Unknown
Nr4a1, nuclear receptor subfamily 4, group A, member 1	NM_010444	8.1	2.90E-02	1.8	5.70E-03	Transcription
Igh-6, immunoglobulin heavy chain 6 (heavy chain of IgM)	BB226392	5.0	2.90E-03	3.2	3.81E-05	Signal transduction/Immune response/Cell proliferation
Ptprf, protein tyrosine phosphatase, receptor type, F	BF235516	4.5	3.69E-03	1.8	1.10E-03	Signal transduction
Pdcd1lg2, programmed cell death 1 ligand 2	NM_021396	4.1	3.79E-05	2.0	2.42E-03	Cell proliferation
Pbx3, pre B-cell leukemia transcription factor 3	NM_016768	3.9	6.23E-06	4.8	1.19E-05	Transcription/Development
Hlf, hepatic leukemia factor	NM_172563	3.4	3.52E-02	6.8	4.03E-04	Transcription/Cell proliferation
Ahr, aryl-hydrocarbon receptor	NM_013464	3.4	2.54E-03	3.1	2.35E-05	Transcription/Signal transduction
Hlx1, H2.0-like homeo box 1	NM_008250	3.4	3.99E-03	1.8	4.96E-03	Transcription
Ier3, immediate early response 3	NM_133662	3.3	9.35E-04	1.5	8.00E-03	Unknown
Erbb2ip, Erbb2 interacting protein	BM240030	3.3	9.17E-04	2.3	1.21E-03	Signal transduction
Tmem71, transmembrane protein 71	AV173260	3.0	4.03E-05	2.5	4.65E-05	Unknown
Pkp2, plakophilin 2	AA516617	3.0	7.13E-06	1.7	1.48E-03	Cell adhesion/Development
Pira1, paired-Ig-like receptor A1	NM_011093	3.0	1.56E-02	2.3	2.13E-02	Cell cycle/Immune response
Tgm2, transglutaminase 2, C polypeptide	BB041811	2.9	2.99E-03	2.9	3.04E-03	Signal transduction/Metabolism/Cell adhesion
Lilrb3, leukocyte immunoglobulin-like receptor, subfamily B, member 3	U96693	2.8	5.50E-03	2.0	8.22E-04	Cell cycle/Immune response
Cables1, Cdk5 and Abl enzyme substrate 1	AF328140	2.8	2.87E-03	1.8	2.59E-02	Cell cycle/Development
H2-DMa, histocompatibility 2, class II, locus DMa	NM_010386	2.8	7.61E-03	2.2	1.74E-02	Immune response/Transport
Ahrr, aryl-hydrocarbon receptor repressor	NM_009644	2.7	9.31E-04	2.2	2.48E-03	Signal transduction/Transcription/Metabolism
Fads3, fatty acid desaturase 3	BE652876	2.7	1.07E-02	1.9	7.20E-03	Metabolism
Tspan6, tetraspanin 6	NM_019656	2.7	1.14E-04	1.7	2.10E-03	Unknown
Tmem51, transmembrane protein 51	BC003277	2.7	5.61E-03	2.1	3.87E-03	Unknown
Rab4a, RAB4A, member RAS oncogene family	NM_009003	2.6	1.41E-03	3.4	1.40E-04	Transport/Signal transduction
Anxa1, annexin A1	NM_010730	2.5	3.65E-03	2.4	4.42E-03	Cell cycle/Cell proliferation/Signal transduction
Pscdbp, pleckstrin homology, Sec7 and coiled-coil domains, binding protein	BC007144	2.4	3.04E-03	1.6	4.34E-02	Cell adhesion
F2rl2, coagulation factor II (thrombin) receptor-like 2	NM_010170	2.4	8.34E-04	2.2	9.01E-04	Coagulation/Signal transduction
RIKEN cDNA C230093N12 gene	BC023470	2.4	2.04E-03	1.5	1.70E-02	Unknown
Cish, cytokine inducible SH2-containing protein	NM_009895	2.4	6.78E-04	1.6	3.19E-02	Cell growth/Signal transduction
Tsc22d1, TSC22 domain family, member 1	BB357514	2.4	2.60E-03	2.0	7.37E-03	Transcription
Wdfy2, WD repeat and FYVE domain containing 2	BB794924	2.4	6.33E-03	1.7	6.51E-03	Unknown
Pld3, phospholipase D family, member 3	NM_011116	2.3	8.47E-03	2.6	4.39E-05	Metabolism
Dnase1l1, deoxyribonuclease 1-like 1	AK009174	2.3	1.84E-02	1.9	2.87E-02	Metabolism
Mylc2pl, myosin light chain 2, precursor lymphocyte-specific	NM_021611	2.3	2.35E-02	2.9	1.82E-03	Unknown
RIKEN cDNA 1700027N10 gene	BC019423	2.3	4.46E-04	2.2	8.76E-04	Unknown
Cyp4f16, cytochrome P450, family 4, subfamily f, polypeptide 16	NM_024442	2.3	5.32E-04	1.9	2.91E-03	Transport
Hoxa5, homeo box A5	BC011063	2.3	7.95E-04	3.9	3.02E-05	Transcription/Development
Crisp3, cysteine-rich secretory protein 3	NM_009639	2.2	1.20E-04	2.1	3.50E-02	Unknown
Procr, protein C receptor, endothelial	NM_011171	2.2	8.99E-03	2.5	5.25E-05	Coagulation
Plek, pleckstrin	AF181829	2.2	5.53E-03	1.8	1.44E-02	Signal transduction
Mitf, microphthalmia-associated transcription factor	BB763517	2.2	9.81E-03	1.9	2.58E-03	Development/Transcription
Metrnl, meteorin, glial cell differentiation regulator-like	BC024445	2.1	2.00E-02	2.2	1.79E-02	Unknown
Prnp, prion protein	BE630020	2.1	2.13E-03	2.3	8.29E-03	Metabolism
Sord, sorbitol dehydrogenase	AV253518	2.1	1.49E-02	2.3	5.31E-04	Unknown
Rpgrip1, retinitis pigmentosa GTPase regulator interacting protein 1	AK015037	2.1	1.98E-04	1.6	3.35E-03	Development
Gsn, gelsolin	NM_010354	2.1	8.22E-03	2.1	2.22E-02	Transport
Hoxa7, homeo box A7	NM_010455	2.0	2.45E-02	1.7	2.38E-02	Transcription/Development
Mef2c, myocyte enhancer factor 2C	AI595932	2.0	2.58E-02	1.7	6.06E-03	Transcription/Development
Eltd1, EGF, latrophilin seven transmembrane domain containing 1	BC017134	2.0	2.94E-04	1.9	4.89E-04	Signal transduction
Flt3, FMS-like tyrosine kinase 3	NM_010229	2.0	2.44E-02	2.0	2.32E-02	Signal transduction/Development
Ncoa1, nuclear receptor coactivator 1	NM_010881	1.9	2.24E-02	1.5	6.81E-03	Transcription/Signal transduction
Ddx4, DEAD (Asp-Glu-Ala-Asp) box polypeptide 4	AK014844	1.9	2.04E-03	13.3	1.92E-06	Development
Calcrl, calcitonin receptor-like	AF209905	1.9	1.37E-02	1.8	1.97E-02	Cell proliferation/Signal transduction/Development
St8sia4, ST8 alpha-N-acetyl-neuraminide alpha-2,8-sialyltransferase 4	NM_009183	1.9	1.87E-03	2.0	1.77E-03	Metabolism
Glul, glutamate-ammonia ligase	AI391218	1.9	1.09E-02	1.7	1.64E-02	Metabolism
Lrp10, low-density lipoprotein receptor-related protein 10	BC011058	1.9	3.27E-03	1.8	5.96E-03	Metabolism/Transport
Hoxa9, homeo box A9	NM_010456	1.8	1.06E-02	2.5	1.33E-03	Transcription/Development
Malat1, metastasis associated lung adenocarcinoma transcript 1	AW012617	1.8	3.31E-02	1.9	1.27E-03	Unknown
Ptk2b, PTK2 protein tyrosine kinase 2 beta	AV026976	1.8	8.99E-03	1.7	1.34E-02	Signal transduction
Igfbp7, insulin-like growth factor binding protein 7	AI481026	1.8	1.25E-02	1.7	2.41E-02	Cell growth/Metabolism
Mxd4, Max dimerization protein 4	BG868949	1.8	1.23E-02	1.8	7.84E-03	Transcription
Sesn1, sestrin 1	BG076140	1.8	1.60E-03	1.8	1.42E-03	Cell cycle
Itm2c, integral membrane protein 2C	NM_022417	1.8	3.67E-03	1.5	2.97E-02	Unknown
RIKEN cDNA 4930504E06 gene	BB010153	1.7	2.71E-02	1.8	2.82E-03	Unknown
Man1a, mannosidase 1, alpha	NM_008548	1.7	9.38E-03	1.6	1.53E-02	Metabolism
Aldoc, aldolase 3, C isoform	BC008184	1.7	2.36E-02	1.8	2.93E-02	Metabolism
Cln3, ceroid lipofuscinosis, neuronal 3, juvenile	NM_009907	1.7	4.55E-03	1.7	5.18E-03	Unknown
Arrb1, arrestin, beta 1	AK004614	1.7	6.77E-03	1.8	7.22E-03	Signal transduction
Cast, calpastatin	AB026997	1.7	8.19E-03	1.8	2.99E-03	Metabolism
Nupr1, nuclear protein 1	NM_019738	1.7	4.92E-03	2.0	8.18E-03	Unknown
Jag2, jagged 2	AV264681	1.7	2.21E-02	1.6	1.15E-02	Signal transduction/Development/Cell proliferation
Bckdha, branched chain ketoacid dehydrogenase E1, alpha polypeptide	NM_007533	1.6	3.33E-02	1.8	1.30E-02	Metabolism/Transcription
Prkcn, protein kinase C, nu	BF160591	1.6	1.17E-02	1.7	3.48E-03	Signal transduction
Pnp, purine-nucleoside phosphorylase	AK008143	1.5	3.02E-03	1.5	2.90E-02	Metabolism
Tcf4, transcription factor 4	AI639846	1.5	2.96E-02	1.5	1.42E-02	Transcription/Development

To confirm the fidelity of the microarray data a subset of 15 genes was selected for validation using quantitative RT-PCR (Table 2). Quantitative real-time RT-PCR was used to measure relative abundances. The housekeeping gene GAPDH was used as an endogenous control to normalize the data. The fold change was calculated between the GFP control and the NA10, ND13 and ND13(N51S) samples respectively. The majority of the individual fold changes determined from the gene array was verified by qRT-PCR (Table 2). Overall the analysis revealed a good correlation between the gene array data and the qRT-PCR results (Pearson correlation, r = 0.83), with a tendency that the PCR results showed greater changes than what the array suggested. Taken together the validation analysis provides confidence in our approach to identify differentially expressed genes with a high likelihood of exhibiting true expression level changes.

**Table 2:** Validation of microarray results with quantitative RT-PCR on unamplified RNA.

*Gene transcript*	*qRT-PCR NA10*	*Array NA10*	*qRT-PCR ND13*	*Array ND13*	*qRT-PCR ND13 N51S*	*Array ND13 N51S*
*Pbx1*	1.7	1.1	2.6	1.3	0.9	1.1
*Pbx2*	0.9	1.3	1.1	1.1	0.6	1.0
*Pbx3*	5.4	5.0	4.2	4.9	0.4	0.8
*HoxA5*	7.5	3.2	7.7	2.1	1.7	0.9
*HoxA7*	1.7	2.1	3.0	2.8	0.5	0.2
*HoxA9*	2.4	1.6	2.9	1.2	0.8	1.0
*HoxB4*	2.9	2.5	1.5	1.4	0.8	1.1
*Meis1*	1.6	1.1	1.7	0.9	1.4	1.2
*Flt3*	5.8	2.2	10.1	2.3	2.1	1.1
*Evi1*	28.1	4.2	8.6	2.9	3.8	1.4
*Anxa1*	3.6	2.0	4.9	2.2	1.0	1.3
*Tgfbi*	2.2	1.8	5.6	2.2	1.7	1.1
*c-Jun*	1.1	1.5	5.3	3.4	1.1	3.4
*Csf2rb*	4.9	2.4	5.6	4.9	1.6	2.2
*Ptprf*	24.5	10.8	100.5	27.2	2.7	2.4

Fold changes are calculated against an empty MIG control and *Gapdh* as endogenous control gene.

### Functional classification and analysis of differentially expressed genes

**Figure 2:**
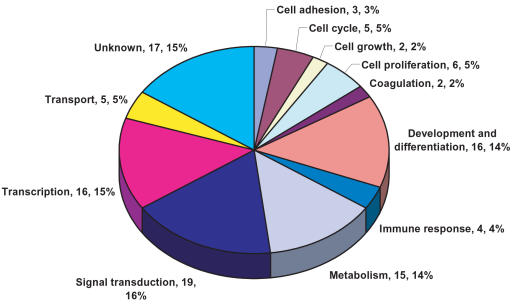
Annotation of differentially expressed genes. Genes that were differentially expressed by both ND13 and NA10 but not the mutant ND13(N51S) mutant were classified according to involvement in different biological processes. Some genes are classified in more than one category resulting in the total number of genes indicated in the figures being greater than the total number of differentially expressed genes.

The 74 genes that were both induced by ND13 and NA10 and whose expression was dependent on an intact homedomain were classified into gene ontology categories according to involvement in different biological processes. The genes were separated into 12 main categories (Figure 2). A relatively high number of differentially expressed genes were classified as being involved in development and differentiation (16 genes), cell cycle, cell growth and/or cell proliferation (13 genes) or signal transduction (19 genes). At least 16 genes were classified as being involved in transcriptional regulation (Table 1). Thus, processes potentially important for cell self-renewal, expansion and transformation.

Several of the genes induced by ND13 and NA10 were Hox or Hox cofactors (*Hoxa5*, *Hoxa7*, *Hoxa9* and *Pbx3*, Table 1). No significant change of gene expression could be seen for *Pbx1*, *Pbx2* or *Meis1*, also verified with Q-RT-PCR (Table 2), but all three genes were expressed in the Sca1+, Lin- BM cell population in concordance with published results [33]. *Meis2* and *Meis3* could not be detected either by gene array or Q-RT-PCR (data not shown). These findings are basically in line what have been reported in other studies [34]–[36]. Besides the Hox or Hox co-factors several other putative target genes of ND13 and NA10 are involved in cell development and proliferation. This included induction of the DEAD-box protein gene *Ddx4*, the hepatic leukemia factor (*Hlf*), the MADS box transcription enhancer factor 2C (*Mef2c*), the prion protein gene (*Prnp*) and Jagged-2 (*Jag2*). The Drosophila ortholog of *Ddx4*, *VASA*, has a central role in germ cell development and is conserved in invertebrates and vertebrates. *Ddx4* is a member of the DEAD box family of ATP-dependent RNA helicases, the same gene family as *Ddx10* belongs to, which has been found in translocations with NUP98 [37]. The prion protein has been shown to be present on human CD34+ bone marrow (BM) stem cells [38] and was recently shown to be expressed on long-term hematopoietic stem cells and to be important in hematopoietic stem cell self-renewal [39]. The *HLF* gene has been found in translocations together with E2A in human acute lymphoblastic leukemia [40] and enforced *HLF* expression has been reported to enhance both HSC engraftment and to inhibit apoptosis [41]. The MEF2 family of regulatory proteins are involved in myogenesis and the *Mef2c* gene is important for normal morphogenesis [42]. Jagged-2 (*Jag2*) is a ligand that activates NOTCH1 and related receptors that are critical for various cell fate decisions. Furthermore, the tyrosine kinase receptor *Flt3* was found to be induced by ND13 and NA10 in Sca1+, Lin- primary BM cells. We and others have previously shown that *Flt3* expression is induced by Meis1 in a context with either high expression of NUP98-Hox fusions [43] or HOXA9 [44] and the present finding further strengthen the conclusion that the *Flt3* is a direct target gene of Hox and Hox co-factors in primary HSCs or progenitor cells.

### Correlation of gene expression in human AML

To discern what target genes identified in the microarray analysis might be involved in leukemic transformation and to investigate if they are associated with Hox and Hox co-factor expression, 34 de nova AML samples collected at diagnosis from adult patients were analyzed. Complete karyotype and FLT3-ITD status was known for all subjects and represented FAB subclass M0-M5 morphologically. Six patients had favorable, 19 had intermediate and 9 had unfavorable cytogenetics (Table 3). Quantitative real-time RT-PCR with TaqMan® Low density array (TLDA) was used to measure gene expression, allowing analysis of all the selected genes in several samples at the same time lowering assay variation and increasing reproducibility. We tested a subset of Hox and Hox cofactor genes, *HOXA5, HOXA7, HOXA9, HOXA10, PBX1, PBX2, PBX3,* and *MEIS1*, and possible Hox target genes *FLT3, HLF, JAG2, MEF2C, DDX4,* and *PRNP*. The gene expressions in leukemia samples were normalized with the housekeeping gene *GUS* as an endogenous control and the expression levels were calculated relative to pooled normal bone marrow (Table 3).

**Table 3:** Gene expression levels in human AML relative to normal bone marrow.

Patient	Cytogenetics	FLT3	FAB	Marrow blasts	*HOXA5*	*HOXA7*	*HOXA9*	*MEIS1*	*PBX1*	*PBX2*	*PBX3*	*MLL*	*FLT3*	*FL*	*HLF*	*JAG2*	*MEF2C*	*PRNP*
1	favorable	ITD+	AML M3	50%	0.0	0.0	0.0	0.1	0.0	2.0	1.1	2.0	22.9	0.3	0.3	0.0	0.4	0.8
2	favorable	Normal	AML M4	22%	0.8	0.0	0.0	3.4	0.0	0.9	2.0	1.5	13.6	0.1	0.0	0.0	12.6	1.8
3	favorable	Normal	AML M3	unknown	0.0	0.4	0.0	0.1	0.0	0.6	0.1	1.1	2.8	0.2	0.3	0.9	0.1	0.4
4	favorable	Normal	AML M3	16%	0.2	0.0	0.0	0.0	0.0	0.3	0.0	0.6	22.7	0.2	0.0	0.0	0.0	0.6
5	favorable	ITD+	AML M3	11%	0.3	0.0	0.0	0.2	0.1	0.2	0.2	1.6	29.5	0.5	0.5	0.4	0.4	1.3
6	favorable	Normal	AML M2	22%	0.3	0.0	0.0	0.1	0.0	0.7	0.7	1.0	9.8	0.3	0.0	0.4	0.6	0.2
7	intermediate	Normal	AML M4	unknown	4.4	17.4	5.5	1.9	0.3	0.8	4.9	2.5	11.1	0.6	1.7	0.2	8.3	1.8
8	intermediate	Normal	AML M1	unknown	7.9	59.2	16.9	8.0	0.1	1.5	3.2	5.3	81.5	0.4	0.8	0.8	16.7	1.6
9	intermediate	Normal	AML M2	50%	15.6	81.8	15.9	20.9	0.0	0.9	9.0	0.9	18.0	0.2	0.2	0.2	1.1	0.9
10	intermediate	ITD+	AML M1	79%	16.0	78.8	24.8	25.4	0.0	1.2	11.6	1.5	37.1	0.2	0.7	0.4	1.3	0.9
11	intermediate	ITD+	AML M2	30%	1.4	7.8	3.3	5.6	0.7	1.6	1.2	2.2	10.5	0.4	1.7	1.2	9.4	0.7
12	intermediate	ITD+	AML M2	55%	38.8	357.5	40.9	65.4	0.1	1.7	17.1	7.0	41.7	3.6	0.8	0.4	14.2	1.7
13	intermediate	Normal	AML M1	87%	1.9	27.7	13.8	17.1	0.5	5.4	2.3	2.0	39.6	0.1	1.5	0.1	0.4	0.6
14	intermediate	ITD+	AML M3	48%	7.7	0.0	0.0	0.1	0.0	0.3	0.9	3.2	37.4	0.4	0.0	0.0	2.0	0.9
15	intermediate	Normal	AML M2	38%	26.2	128.1	30.0	22.6	0.0	0.7	12.1	8.1	50.8	4.2	0.8	0.4	5.7	2.6
16	intermediate	Normal	AML M1	46%	0.2	0.9	0.1	0.2	0.1	1.8	0.3	2.8	8.4	0.2	0.5	0.2	2.1	0.6
17	intermediate	Normal	AML M1	58%	26.1	92.1	36.6	61.5	0.3	0.9	7.7	1.8	38.2	0.5	0.8	2.0	0.4	0.6
18	intermediate	ITD+	AML M2	77%	15.3	58.9	20.7	12.7	0.0	0.9	1.8	2.7	41.4	0.2	1.4	0.8	0.8	0.5
19	intermediate	Normal	AML M4	40%	27.7	120.7	22.2	25.3	0.0	1.0	6.5	1.9	30.1	0.8	0.0	0.4	7.8	2.0
20	intermediate	Normal	AML M0	90%	1.1	1.4	0.4	0.8	0.7	1.4	0.7	0.7	3.4	0.5	0.7	0.3	0.6	0.8
21	intermediate	Normal	AML M1	70%	1.1	20.0	2.4	13.0	0.0	1.5	0.0	2.8	59.3	0.4	0.2	0.4	3.5	1.4
22	intermediate	ITD+	AML M1	74%	20.5	45.1	44.9	42.9	0.0	0.3	3.8	3.4	81.4	0.0	0.1	0.8	0.9	0.7
23	intermediate	Normal	AML M1	59%	0.0	0.6	0.0	0.1	0.0	0.9	1.5	4.1	55.6	1.2	0.2	0.1	1.6	1.4
24	intermediate	ITD+	AML M1	90%	19.0	184.9	34.7	61.1	0.0	0.4	11.9	2.4	40.2	0.4	2.2	0.4	4.3	0.8
25	intermediate	Normal	AML M1	63%	15.3	40.9	21.6	12.0	0.3	1.6	2.8	5.6	55.0	0.9	0.0	0.6	9.9	0.8
26	unfavorable	Normal	AML M2	27%	11.9	96.9	11.3	5.8	1.2	2.1	1.8	4.3	44.3	0.7	14.7	0.7	29.2	1.7
27	unfavorable	Normal	AML M5	74%	1.9	10.3	2.3	1.0	0.1	5.3	5.3	1.8	34.2	0.5	1.5	0.3	17.7	1.9
28	unfavorable	Normal	AML M4	75%	4.2	37.2	10.4	10.1	0.0	0.3	0.8	0.8	44.7	0.4	0.9	1.0	0.1	0.8
29	unfavorable	Normal	AML M5	20%	7.6	27.8	7.9	13.6	0.1	0.7	4.2	1.4	12.4	0.5	0.4	0.1	5.0	1.3
30	unfavorable	Normal	AML M4	59%	2.4	0.1	0.0	7.9	0.1	0.9	1.6	3.3	33.1	0.2	0.0	0.0	14.8	1.8
31	unfavorable	Normal	AML M2	64%	11.3	58.1	14.2	11.3	0.3	0.3	6.2	5.9	35.4	0.9	0.5	0.7	9.1	2.0
32	unfavorable	Normal	AML M1	43%	3.8	12.9	9.0	6.4	0.4	5.7	2.8	7.4	87.3	1.0	1.7	2.2	7.5	1.2
33	unfavorable	Normal	AML M1	85%	11.5	90.2	28.7	7.2	0.2	3.2	0.2	7.3	118.4	0.7	5.8	7.3	8.3	2.1
34	unfavorable	Normal	AML M1	78%	2.7	18.0	4.5	4.0	0.1	3.5	0.3	2.3	27.5	0.1	0.4	0.9	2.8	1.5

**Figure 3:**
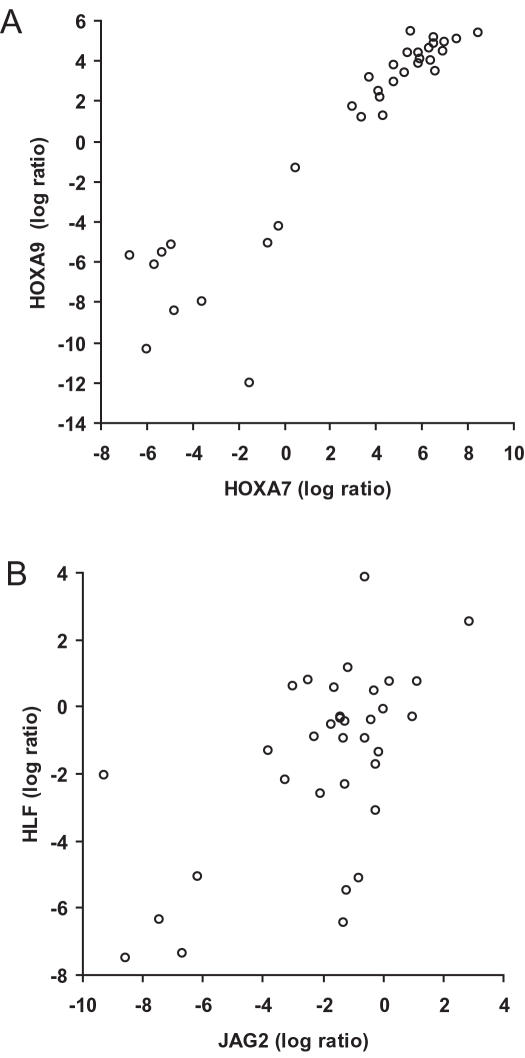
Correlation between HOXA7 and HOXA9 (A) and JAG2 and HLF (B) gene expression in human AML analyzed with TaqMan Low Density Array (TLDA). Spearman rank correlation analysis was done on the log ratio values obtained from the TLDA assay and calculated with the 2^−ΔΔCT^ method (n = 34).

Two of the selected genes could not be detected in the majority of the samples in either normal or leukemia bone marrow (*HOXA10 and DDX4*) and were therefore excluded from further analysis. Analysis of the selected Hox A cluster and Hox cofactor genes revealed a high degree of co-expression between these in leukemia samples, where correlation coefficient between *HOXA5, HOXA7, HOXA9* and *MEIS1* ranged between 0.83 and 0.92 (Spearman rank correlation, p<0.0001 in all cases, e.g. in Figure 3A). *PBX3* gene expression also correlated with these (r = 0.67–076, p<0.0001) but *PBX2* and *PBX1* did not. Furthermore, patients with intermediate or unfavorable cytogenetics had significantly higher expression of *MEIS1*, *PBX3*, *HOXA5*, *HOXA7* and *HOXA9* compared to patients with favorable cytogenetics (non-parametric Kruskal-Wallis ANOVA, e.g. *HOXA7* p = 0.0013 and p = 0.018 respectively) but there was no significant difference between patients with intermediate and unfavorable cytogenetics. These findings suggest a tight co-regulation of these factors and define a subset of homeodomain transcription factors linked to leukemic development and are in concordance with other reports [45], [46].

Among the selected possible direct target genes, *FLT3* gene expression showed the closest association with *HOXA5*, *HOXA7*, *HOXA9* and *MEIS1* (Spearman, r-value between 0.45–0.57, p–value 0.016–0.0020) but no significant correlation was seen with *PBX3* expression (r = 0.22, p = 0.20). Furthermore, *FLT3* expression showed a weak but significant correlation with bone marrow blast counts (r = 0.36, p = 0.049). Moreover, *FLT3* expression was significantly higher in patients with intermediate or unfavorable cytogenetics (Kruskal-Wallis, p = 0.047 and p = 0.046 respectively) but there was no significant association between *FLT3* expression and FLT3-ITD status (p = 0.74). Thus, *FLT3* expression was associated with *HOXA5*, *HOXA7*, *HOXA9* and *MEIS1* in human leukemia and this gene expression profile was confined to leukemia with either intermediate or unfavorable cytogenetics.

Expression of the *HLF* gene correlated significantly with *HOXA7* and *HOXA9* expression (Spearman, r = 0.46, p = 0.0060 and r = 0.43, p = 0.011 respectively), but not with *HOXA5* (r = 0.24, p = 0.17), *MEIS1* (r = 0.25, p = 0.15) or *PBX3* (r = 0.25, p = 0.16). The same association was seen for *JAG2*, with a positive correlation with *HOXA7* and *HOXA9* gene expression (Spearman, r = 0.46, p = 0.0062 and p = 0.0068, respectively) but not with *HOXA5*, *MEIS1* or *PBX3* (r = 0.31, p = 0.074, r = 0.32, p = 0.061 and r = −0.02, p = 0.98 respectively). Furthermore, *JAG2* and *HLF* gene expression correlated (r = 0.47, p = 0.0052, Figure 3B) and there was also a significant correlation between *JAG2* and *FLT3* expression (r = 0.39, p = 0.024). Altogether, these findings suggest that HOXA7 and HOXA9 could be directly involved in *JAG2* and *HLF* gene regulation and possibly, unlike *FLT3,* independent of MEIS1.

The expression of the *MEF2C* and *PRNP* genes showed a tight co-association (r = 0.70, p<0.0001) and both showed significantly higher expression in patients with unfavorable vs. favorable cytogenetics (Kruskal-Wallis ANOVA, p = 0.017 and p = 0.026 respectively). However, their expression did not correlate with *HOXA5*, *HOXA7, HOXA9* or *MEIS1* and only weakly with *PBX3* expression in human leukemia (Spearman, r = 0.37, p = 0.033 and r = 0.44, p = 0.078 respectively). This suggests that their induction may be restricted to NUP98-Hox fusions and that they are not regulated by the native HOXA5, HOXA7, HOXA9 or MEIS1 in human bone marrow cells.

## Discussion

The goal of this study was to identify gene expression changes that may underlie the potent growth promoting effects of Hox on primitive hematopoietic cells. Our strategy included use of two NUP98-Hox fusions with strong overlapping functional effects versus a functionally “dead” mutant form coupled with analysis of early induced gene expression changes in a HSC/progenitor cell enriched fractions. Key results included the identification of a limited number of induced genes mainly involved in cell development, cell proliferation and signal transduction, consistent with the potent effects of these fusions on promoting primitive hematopoietic cell expansion and differentiation block *in vitro* and their ability to collaborate in leukemic transformation [19], [21], [27]. Among the identified genes are several intriguing candidates as HSC regulators and/or possible leukemogenic targets e.g. the tyrosine receptor *Flt3*, the prion protein *Prnp*, the transcription factor *Hlf* and the Notch ligand *Jag2*. Moreover, induction of *Hoxa5*, *Hoxa7*, *Hoxa9* and *Pbx3* by ND13 and NA10 was observed in HSC/progenitor cells that may define a minimal Hox transforming profile that seems to be shared with other NUP98-Hox and MLL fusions. Furthermore, *FLT3*, *HLF* and *JAG2* expression correlated with Hox genes in human AML that both confirms the fidelity of the microarray analysis and suggests the importance of these genes for Hox genes ability to trigger HSC expansion and to serve as a first step in leukemic transformation.

Importantly, several of the suggested target genes reported herein overlap with those recently published by the study of Chung et al., in which human CD34^+^ cord blood was used to investigate the effects induced by *NUP98-HOXA9*. These included the *HOXA5*, *HOXA7*, *HOXA9* and *PBX3* genes but perhaps more intriguing also *HLF*. Elevated *HLF* expression can both enhance HSC engraftment and inhibit apoptosis [41]. The homeodomain dependent induction of *Hlf* expression by ND13 and NA10 may explain the proliferative advantage induced by these genes on primary HSC and progenitor cells *in vitro* and the high level expansion of long term repopulating cells induced NA10 [19], [27]. The finding that *HLF* also correlated with *HOXA7* and *HOXA9* in human leukemia suggests that this gene also might be important for Hox induced cell transformation and development of leukemia.

The finding of homeodomain dependent induction of *Jag2* by ND13 and NA10 in Sca1^+^, Lin^−^ BM cells, and that *JAG2* expression correlated with *HOXA7* and *HOXA9* expression in AML samples is also very interesting since *JAG2* was recently found to be overexpressed in CD34(+)CD38(−) isolated leukemic stem cells from AML patients [47]. Moreover, Serrate, the Drosophila homologue of JAG2, has been identified as a component of Hox-dependent pathways [48] and in C. elegans the Hox protein LIN-39 and its Pbx-like cofactor CEH-20 are required for LIN-12/Notch-mediated signaling and for the expression of the genes encoding the LIN-12/Notch receptor and its ligand LAG-2/Delta/Serrate [49]. Thus, the Notch signaling pathway might be part of the effects induced by Hox and Hox co-factors.

Calvo et al. have reported that NUP98-HOXA9 enforce strong transcription of endogenous *Hoxa9* and *Hoxa7,* which further strengthen that different NUP98-Hox fusions have common target genes. HOXA9 is frequently induced in human AML with poor prognosis [50] and Hoxa9 can induce leukemia in murine BM transplantation models in collaboration with Meis1 [51] similar to what we have shown for ND13 and NA10 [19], [21]. Furthermore, mixed-lineage-leukemia (MLL) fusion genes, induce a characteristic pattern of Hox A cluster genes, including *Hoxa7* and *Hoxa9* in myeloid cells [52]. Both these genes are required for efficient *in vitro* myeloid immortalization by MLL-ENL and in a bone marrow transplantation model Hoxa9 is essential for MLL-dependent leukemogenesis *in vivo* [52]. Furthermore, deregulation of *FLT3* or *FLT3* mutations are frequently found in AML [53]. Meis1 was recently shown to directly induce *Flt3* expression in murine BM cells together with either Hoxa9 or ND13 or NA10 [43], [44] and we have also shown that high *Flt3* expression is sufficient to induce AML transformation in mice using preleukemic BM cells expressing either ND13 or NA10 [43]. Interestingly, *Flt3* has also been reported to be expressed together with *Pbx3*, *HoxA7*, *HoxA9* and *Meis1* in a gene expression profile induced by MLL-ENL [54], thus similar to what we observed for ND13 and NA10 in this study. In addition, our results reveal an association between *HOXA5*, *HOXA7*, *HOXA9*, *MEIS1* and *FLT3* expression in human AML. In conclusion these findings indicate that induction of *Flt3*, *Hoxa7, Hoxa9* and *Pbx3* and possibly other Hox genes (i.e. *Hoxa5*) in part underlie the transforming effects of ND13 and NA10 and perhaps support a common mechanism for leukemogenesis triggered by both NUP98 and MLL fusion genes.

Finally, the gene array results also indicated that the NUP98-Hox genes act principally as strong transcriptional activators. Importantly, the set of genes that showed overlap between ND13 and NA10 were all induced genes suggesting that gene transcription activation rather then repression is the key to their functional effects on primitive hematopoietic cells. Interestingly, in the study by Ghannam et al. where HOXA9 or NUP98-HOXA9 were expressed in myeloid cell lines, the majority of the genes showed predominant induced expression [55]. Furthermore, NUP98-HOXA9 affected about eight times more genes than HOXA9, with a substantial number of them regulated by the fusion but not by the native HOXA9 protein, intriguingly including the *Mef2c* gene. The finding that the *Mef2c* gene was induced also by both ND13 and NA10, but did not correlate with Hox or Hox co-factor expression in human AML samples indicate that this gene, plus the prion protein, *Prnp*, gene could be novel direct targets of these NUP98-Hox fusions and not normally effected by native *HOXA5*, *HOXA7*, *HOXA9* or *MEIS1* genes. However, other Hox genes not included in our analysis could of course still be involved in their regulation. In conclusion our findings support that NUP98-Hox fusion proteins are aberrant transcriptional activators whose activity depends on the DNA binding homeodomain but also has stronger and wider transcriptional effects than the native Hox protein. Findings by Chung et al. provide evidence that this effect, at least in part, is mediated by decreased susceptibility to CUL-4A-dependent ubiquitination of NUP98-Hox fusions increasing their protein half-lives [56].

In summary this study identify gene expression changes that involve several different biological processes important for HSC self-renewal and proliferation and point to several interesting genes suggesting that NUP98-Hox fusions target multiple mechanisms that could potentially explain how they both transform and induce expansion of HSCs, which in turn may lead to leukemia. The next step in our investigation will be to elucidate their relative role in these processes in animal models for HSC expansion and for transforming activity. These results will also be of great help in ongoing efforts for genome wide analysis of Hox binding sites and for investigating direct binding of Hox and Hox co-factors to regulator sequences.
